# Construction and Performance Evaluation of Zein/PVDF Electrospun Nanofiber Membranes as Functional Carriers for Food Packaging

**DOI:** 10.3390/foods15162909

**Published:** 2026-08-20

**Authors:** Fei Yao, Yishuang Dong, Chan Jin, Zihao Li, Changhong Liu, Fusheng Chen

**Affiliations:** 1 School of Food and Biological Engineering, Engineering Research Center of Bio-Process, Hefei University of Technology, Hefei 230601, Anhui, China; 2 College of Food Science and Technology, Henan University of Technology, Zhengzhou 450001, Henan, China

**Keywords:** Zein, poly(vinylidene fluoride), bromothymol blue, electrospinning, freshness monitoring, intelligent packaging

## Abstract

This study aimed to systematically evaluate the effects of the Zein/poly(vinylidene fluoride) (PVDF) blend ratio on the formation, structure, and performance of electrospun nanofiber membranes and to further assess the feasibility of incorporating bromothymol blue (BTB) into the optimized matrix as a pH-responsive functional component for intelligent food-packaging applications. Increasing PVDF content reduced the conductivity but increased the viscosity of the spinning solutions, and all formulations exhibited shear-thinning behavior. Pure Zein failed to form continuous fibers, whereas PVDF incorporation promoted uniform fibrous networks, with average fiber diameters increasing from 113.02 ± 27.06 nm to 530.68 ± 113.33 nm. FTIR and TGA/DTG analyses confirmed the coexistence of Zein and PVDF and the improved thermal stability associated with increasing PVDF content. Surface hydrophobicity and water resistance increased with PVDF content, whereas water vapor permeability (WVP) and water solubility (WS) increased with the Zein proportion. Considering spinnability, morphology, mechanical behavior, barrier performance, and water stability, the Zein/PVDF = 5:5 formulation provided a comparatively balanced performance and was selected as the functional carrier matrix. Incorporation of BTB into this matrix produced a distinct pH-responsive color change, and the color difference (ΔE) showed a strong correlation with shrimp pH during storage (R^2^ = 0.997), demonstrating the feasibility of the optimized membrane as a freshness-responsive functional carrier.

## 1. Introduction

Electrospinning is an efficient method for fabricating fibers ranging from nanoscale to microscale. It has attracted substantial attention because of its simple processing, operational flexibility, low cost, and broad material compatibility [1,2]. Compared to conventional film-forming methods, electrospun nanofiber membranes typically exhibit a large specific surface area, excellent pore interconnectivity, high porosity, and tunable morphologies. These structural features facilitate the encapsulation and controlled release of active substances, while enhancing interfacial interactions and functional responsiveness. In addition, bioactive compounds can be directly incorporated into the spinning solution, imparting multifunctional properties to the resulting membranes, including antioxidant, antibacterial, and sustained-release activities [3,4,5]. Accordingly, electrospinning has been widely applied in food packaging, tissue engineering, wound dressing, and drug delivery, particularly in the design and fabrication of functional bio-based materials [6,7].

With the growing interest in sustainable materials, proteins have emerged as important electrospinning feedstocks because of their availability, biocompatibility, and biodegradability [8,9]. However, protein-based nanofibers often exhibit limited molecular-chain entanglement, insufficient mechanical strength, and poor stability in aqueous environments [10,11,12]. Zein, a major corn protein with favorable film-forming ability, has been widely used as a carrier for active compounds, although these limitations restrict its use as a stand-alone fibrous matrix. Blending Zein with polymers possessing complementary properties therefore represents an effective strategy for improving fiber formation and membrane stability. For example, poly(ethylene oxide) (PEO) has been used to regulate the rheological properties and electrospinnability of Zein solutions [13], while Zein/poly(vinyl alcohol) (PVA) systems have also been explored for active packaging applications [14]. Previous studies have further demonstrated the incorporation of essential-oil inclusion complexes, plant extracts, and antibacterial components into Zein-based electrospun systems [15,16,17], highlighting the potential of Zein as a functional carrier.

Poly(vinylidene fluoride) (PVDF) is a semicrystalline fluoropolymer with favorable electrospinning processability, hydrophobicity, thermal and chemical stability, and mechanical integrity [18,19]. These properties can complement the limitations of Zein, particularly in terms of fiber formation and stability under humid or aqueous conditions. The feasibility of combining these two polymers has been demonstrated by Teng et al. [20], who prepared Zein/PVDF micro/nanofibers and reported improved mechanical performance after PVDF incorporation. Nevertheless, the effects of the Zein/PVDF composition on spinning-solution behavior, fiber formation, membrane structure, and packaging-related properties have not been systematically elucidated. Because variations in the Zein/PVDF ratio can simultaneously affect conductivity, rheological behavior, jet stability, fiber morphology, surface characteristics, water-related properties, and mechanical performance, systematically evaluating these composition-dependent changes is important for understanding the structure–property relationships of the Zein/PVDF electrospun system.

Accordingly, Zein/PVDF electrospun membranes with different blend ratios were prepared under identical processing conditions, and their solution properties, rheological behavior, fiber formation, structural characteristics, thermal stability, wettability, water vapor permeability, water solubility, and mechanical performance were systematically evaluated. This study aimed to establish the composition–solution–fiber–structure–performance relationship and identify a comparatively balanced carrier matrix for subsequent functionalization. Bromothymol blue (BTB) was subsequently incorporated as a model pH-responsive component to evaluate the feasibility of the optimized fibrous matrix for freshness monitoring.

## 2. Materials and Methods

### 2.1. Reagents

Zein (Z3625) was purchased from Sigma-Aldrich (Shanghai, China), and poly(vinylidene fluoride) (PVDF, 761) was obtained from Arkema (Puteaux, France). N,N-Dimethylformamide (DMF) and acetone, both of analytical grade, were purchased from Tianjin Fuyu Fine Chemical Co., Ltd. (Tianjin, China). Bromothymol blue (BTB) was obtained from Shanghai Macklin Biochemical Co., Ltd. (Shanghai, China). Anhydrous calcium chloride (CaCl_2_, analytical grade) was supplied by Tianjin Kemiou Chemical Reagent Co., Ltd. (Tianjin, China). Phosphate-buffered saline (PBS) powder was purchased from Beijing Solarbio Science & Technology Co., Ltd. (Beijing, China).

### 2.2. Experimental Methods

#### 2.2.1. Preparation of Zein/PVDF Nanofiber Membranes

##### Preparation of Zein/PVDF Spinning Solutions

Zein and PVDF were weighed to obtain mass ratios of 10:0, 7:3, 5:5, 3:7, and 0:10, while the total polymer concentration was fixed at 15% (*w*/*v*) for all the samples. The polymers were dissolved in a mixed solvent of DMF and acetone (3:2, *v*/*v*) and magnetically stirred at 45 °C until homogeneous spinning solutions were obtained. The solutions were then allowed to stand for 30 min to remove trapped air bubbles before electrospinning.

##### Electrospinning of Zein/PVDF Nanofiber Membranes

**Figure 1:**
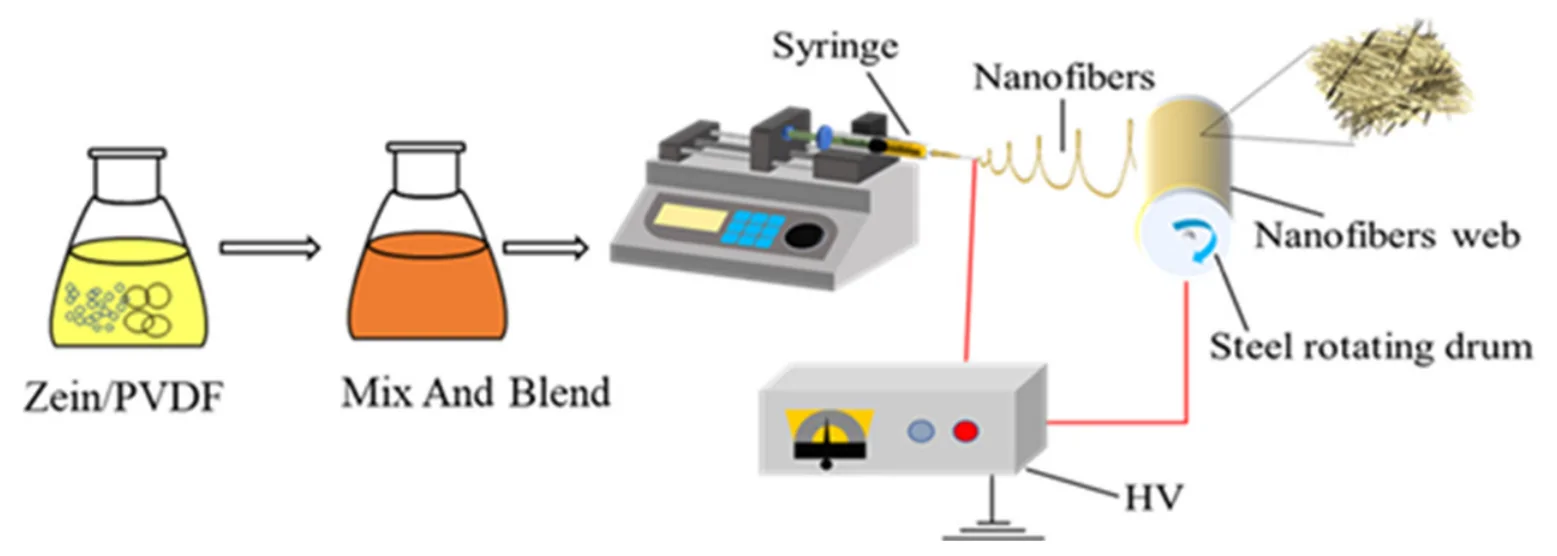
Schematic illustration of the electrospinning process.

Each spinning solution was loaded into a syringe fitted with a 23-gauge stainless-steel needle. Electrospinning was carried out using an electrospinning system (E05, Foshan Light Industry Precision Measurement and Control Technology Co., Ltd., Foshan, China). The processing conditions were set as follows: flow rate of 0.5 mL/h, applied voltage of 18 kV, tip-to-collector distance of 15 cm, ambient temperature of 25 °C, and relative humidity of 50 ± 5%. During electrospinning, the spinneret was moved laterally in a reciprocating manner at a traverse speed of 20 cm/min, and the fibers were collected on a rotating drum operating at 400 rpm, as illustrated in Figure 1. After electrospinning, the membranes were vacuum-dried at 50 °C for 12 h to remove residual solvent and subsequently sealed for storage.

### 2.3. Determination of Solution Properties

The surface tension of the spinning solutions was determined at 25 ± 1 °C using a surface tension meter (DSA25, Krüss, Hamburg, Germany), and each formulation was analyzed in triplicate. The electrical conductivity was measured with a conductivity meter (DSS-11A, Shanghai Jingci Instrument Co., Ltd., Shanghai, China) after calibration with a standard solution. The readings were recorded once stable, and the results are expressed in μS/cm. Rheological measurements were performed at 25 °C using a HAAKE rheometer (RS-6000, Thermo Fisher Scientific, Waltham, MA, USA) equipped with a parallel-plate geometry with a plate diameter of 25 mm and a gap of 0.2 mm. The apparent viscosity was recorded over a shear rate range of 0.1–1000 s^−1^.

### 2.4. Morphological Observation of Nanofiber Membranes by SEM

The electrospun nanofiber membranes were cut into small pieces, affixed to sample stubs, and coated with gold sputter coating prior to viewing. The surface morphologies of the Zein/PVDF nanofiber membranes with different mass ratios were examined using a scanning electron microscope (SEM, TESCAN MIRA LMS, Brno, Czech Republic) operating at an accelerating voltage of 5 kV and magnification of 5000×. Finally, the acquired SEM images were processed using Image-Pro Plus 6.0 software.

### 2.5. Fourier Transform Infrared Spectroscopy (FTIR) Analysis

The chemical composition of the nanofiber membranes was characterized using FTIR spectroscopy (Nicolet iS20, Thermo Fisher Scientific, Waltham, MA, USA) equipped with an ATR accessory. Spectra were acquired from 4000 to 400 cm^−1^ at a resolution of 4 cm^−1^, and each spectrum was averaged over 32 scans.

### 2.6. X-Ray Diffraction (XRD) Analysis

The crystalline structure of pure Zein powder, PVDF, and Zein/PVDF composite nanofibers with varying mass ratios was examined by X-ray diffraction (XRD; SmartLab SE, Rigaku, Tokyo, Japan). XRD measurements were carried out with Cu Kα radiation (λ = 1.54056 Å) at an operating voltage of 40 kV and a current of 40 mA. The diffraction profiles were recorded over a 2θ range of 5–90° at a scanning rate of 10° min^−1^.

### 2.7. Thermal Stability Analysis

The thermal behavior of Zein/PVDF nanofiber membranes with varying mass ratios was assessed using a thermogravimetric analyzer (TGA; Q600, TA Instruments, New Castle, DE, USA). For each measurement, approximately 10 mg of the membrane sample was loaded into the instrument and heated from 35 to 600 °C at 10 °C/min under a nitrogen atmosphere, with the flow rate maintained at 20 mL/min.

### 2.8. Water Contact Angle, Water Solubility, and Water Vapor Permeability

The water contact angles of the Zein/PVDF nanofiber membranes with different mass ratios were measured using a contact angle tester (DSA25, KRÜSS, Hamburg, Germany). The membrane samples were cut into 2 × 5 cm pieces and placed on a sample stage. After the contrast and brightness were adjusted appropriately, measurements were conducted under standard ambient conditions.

Water solubility (WS) was determined using a gravimetric method with minor modifications from a previously reported edible-film water-solubility procedure [21]. The membrane samples were dried to a constant weight, and the initial dry weight was recorded as W_0_. The samples were then immersed in distilled water for 24 h, removed, and dried again to a constant weight. The remaining dry weight was recorded as W_1_, and WS was calculated as shown in (1).

(1) $$WS\;(\%)=[(W_{0}-W_{1})\;/\;W_{0}]\times 100$$

Water vapor permeability (WVP) was determined using a gravimetric cup method with minor modifications from the procedure reported by McHugh et al. [22]. Anhydrous calcium chloride was placed in the permeation cup, and the membrane sample was sealed over the cup opening. The cup was stored at 25 °C and 50% RH, and its mass change was recorded at regular intervals. WVP was calculated from the mass gain as shown in Equation (2).

(2) $$WVP(g\;mm/m^{2}hkPa)=\frac{\Delta m\times L}{\;A\times t\times \Delta P}\;$$

where Δ*m* is the mass change in the permeation cup, *L* is the membrane thickness, A is the effective permeation area, *t* is the testing time, and ΔP is the water vapor pressure difference across the membrane. Under the test conditions of 25 °C and 50% RH, Δ*P* was calculated as 1.58 kPa.

### 2.9. Mechanical Properties Measurement

The mechanical properties of the Zein/PVDF nanofiber membranes were determined using a universal testing machine (AGX-100N, Shimadzu, Kyoto, Japan). The membranes were cut into rectangular strips (8 cm × 1 cm), and the thickness of each specimen was measured and averaged. Tensile tests were performed with an initial gauge length of 5 cm, pretension of 100 cN, and tensile rate of 1 mm/min. At least five specimens were tested for each formulation in every batch. The tensile strength and elongation at break were calculated from the resulting stress–strain curves.

### 2.10. Preparation and pH-Responsive Performance Evaluation of BTB/Zein/PVDF Indicator Membranes

A predetermined amount of bromothymol blue (BTB) was dissolved in a DMF/acetone mixed solvent (3:2, *v*/*v*) and ultrasonically dispersed in the dark for 15 min to obtain a homogeneous BTB solution. Zein and PVDF were then added at the optimized mass ratio of 1:1, with a total polymer concentration of 15% (*w*/*v*), and the mixture was magnetically stirred at 45 °C for 12 h until homogeneous. The BTB/Zein/PVDF indicator membrane was prepared using the electrospinning conditions described above and stored in a sealed package protected from light after drying. To evaluate pH-responsive behavior, the indicator membranes were cut into circular discs with a diameter of 1.0 cm and individually immersed in 10 mL of PBS solutions adjusted to pH 5–12. After exposure for 15 min at approximately 25 °C, the membrane color changes were recorded, and the corresponding UV–vis absorption spectra were measured over 350–750 nm using a UV–vis spectrophotometer (UV-2700i, Shimadzu, Kyoto, Japan). In a separate freshness-monitoring experiment, approximately 10 g of shrimp was placed in each storage container, and three independent containers were prepared for each experimental group. A BTB/Zein/PVDF indicator membrane was fixed to the inner surface of the container lid above the shrimp samples without direct contact. The containers were stored at 25 °C, and shrimp pH and membrane color changes were recorded for all three replicates at 0, 6, 12, 24, 36, and 48 h. The L*, a*, and b* values of the indicator membranes were measured using a colorimeter (LS173, Shenzhen Linshang Technology Co., Ltd., Shenzhen, China), and the total color difference (ΔE) was calculated using the membrane color at 0 h as the reference, as shown in Equation (3).

(3) $$\Delta E=\sqrt{{\left(L^{*}-L_{0}^{*}\right)}^{2}+{\left(a^{*}-a_{0}^{*}\right)}^{2}+{\left(b^{*}-b_{0}^{*}\right)}^{2}}$$

where $L_{0}^{*}$, $a_{0}^{*}$ and ${\;b}_{0}^{*}$ represent the lightness, red–green value, and yellow–blue value of the indicator membrane at 0 h, respectively, while $L^{*}$, $a^{*}$, and $b^{*}$ represent the corresponding chromatic parameters of the indicator membrane at different storage times.

### 2.11. Statistical Analysis

Data are expressed as the mean ± standard deviation (SD). Statistical differences among groups were analyzed by one-way analysis of variance (ANOVA), followed by Duncan’s multiple range test using SPSS 20.0. Differences were considered statistically significant at *p* < 0.05. All graphs were prepared using Origin 2021 software.

## 3. Results

### 3.1. Subsection

#### 3.1.1. Analysis of Solution Properties

**Figure 2:**
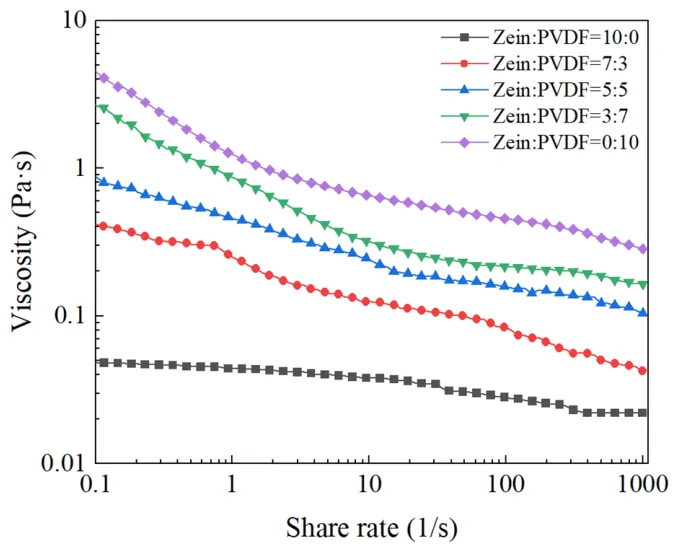
Logarithmic plots of viscosity versus shear rate for Zein/PVDF blend solutions with different mass ratios.

The physicochemical properties of the Zein/PVDF spinning solutions are summarized in Table 1. Increasing the PVDF fraction resulted in a pronounced decrease in electrical conductivity, from 430 ± 0.70 μS/cm for the Zein/PVDF = 10:0 solution to 8.95 ± 0.03 μS/cm for pure PVDF, with significant differences among the formulations (*p* < 0.05). This trend can be attributed to the greater abundance of ionizable and polar groups in Zein, whereas PVDF contains fewer groups capable of contributing mobile charge carriers. Because solution conductivity influences the charge density and electrostatic stretching of the jet during electrospinning, the marked decrease in conductivity with increasing PVDF content is expected to contribute to subsequent changes in fiber formation and diameter [23,24]. In contrast, the surface tension varied only within a narrow range of 37.59–39.58 mN/m, and no significant differences were observed among the formulations (*p* > 0.05). The limited variation suggests that surface tension was governed predominantly by the common DMF/acetone solvent system rather than by the Zein/PVDF ratio [23,24]. Accordingly, the pronounced changes in conductivity, together with the composition-dependent rheological behavior shown in Figure 2, appear to have played a more important role than surface tension in regulating electrospinning behavior and subsequent fiber morphology.

**Figure 3:**
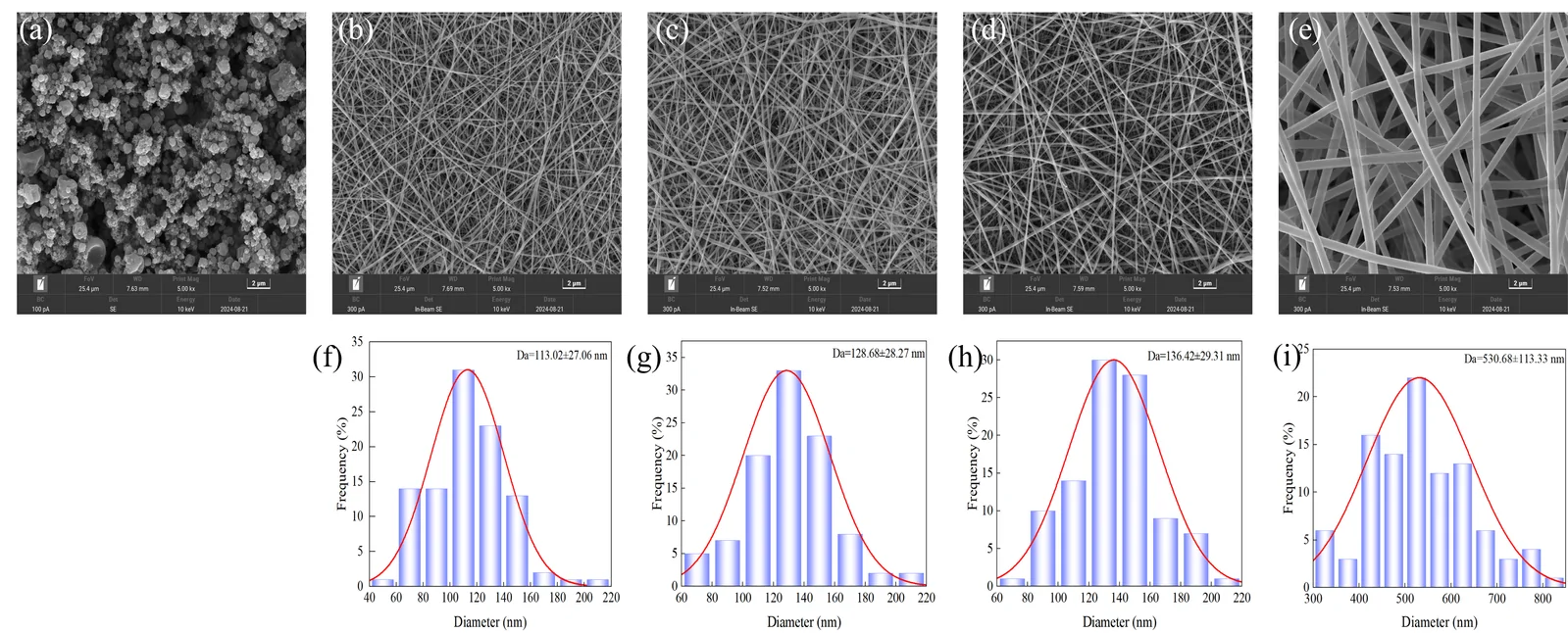
SEM micrographs and corresponding fiber diameter distributions of Zein/PVDF electrospun membranes with different mass ratios: (**a**) Zein/PVDF = 10:0; (**b**) Zein/PVDF = 7:3; (**c**) Zein/PVDF = 5:5; (**d**) Zein/PVDF = 3:7; (**e**) Zein/PVDF = 0:10; (**f**) fiber diameter distribution of Zein/PVDF = 7:3; (**g**) fiber diameter distribution of Zein/PVDF = 5:5; (**h**) fiber diameter distribution of Zein/PVDF = 3:7; and (**i**) fiber diameter distribution of Zein/PVDF = 0:10. No fiber diameter distribution was calculated for the Zein/PVDF = 10:0 sample because a continuous fibrous structure was not formed under the selected electrospinning conditions.

Figure 2 shows the apparent viscosities of the Zein/PVDF blended solutions with different mass ratios as a function of shear rate. The apparent viscosity increased progressively as the Zein content decreased and the PVDF content increased, with the lowest value observed for the 10:0 formulation and the highest value for the 0:10 formulation. All samples exhibited a decrease in viscosity with increasing shear rate, demonstrating typical shear-thinning behavior and non-Newtonian flow characteristics [25]. This behavior was mainly associated with the entanglement and orientation of polymer chains under shear. As the shear rate increased, molecular chains gradually aligned along the shear direction and the entangled network was partially disrupted, resulting in a decrease in apparent viscosity. With increasing PVDF content, the blended system exhibited stronger polymer-chain entanglement and higher viscosity, whereas a high Zein proportion provided insufficient chain entanglement to maintain a stable polymer network. These rheological differences are closely related to electrospinning behavior: enhanced chain entanglement at higher PVDF contents promoted jet stability and continuous fiber formation, while insufficient entanglement in the Zein-rich system increased the tendency toward bead formation, particulate structures, and fiber discontinuity. This interpretation is consistent with the morphology observed in Figure 3.

#### 3.1.2. Morphology and Fiber Diameter Distribution of Zein/PVDF Nanofiber Membranes

Figure 3 shows a clear composition-dependent evolution of the electrospun morphology. The pure Zein formulation (Zein/PVDF = 10:0) failed to develop a continuous fibrous network and instead exhibited predominantly particulate or aggregated structures, indicating that the molecular entanglement of the Zein solution was insufficient to sustain stable fiber formation under the selected electrospinning conditions. Following the incorporation of PVDF, continuous and interconnected fibrous networks progressively developed, accompanied by improved fiber continuity and fewer bead-like defects. This morphological transition is consistent with the rheological results shown in Figure 2, where increasing PVDF content resulted in higher solution viscosity and stronger polymer-chain entanglement, thereby improving jet stability during electrospinning. Among the fiber-forming samples, the average fiber diameter increased from 113.02 ± 27.06 nm to 530.68 ± 113.33 nm as the PVDF fraction increased. This trend can be interpreted from the combined changes in viscosity and conductivity. Increased viscosity and chain entanglement resist excessive jet thinning, whereas the marked reduction in conductivity decreases charge-induced stretching of the electrospinning jet. In comparison, the relatively small variation in surface tension suggests that it played a more limited role in determining fiber diameter. Taken together, the results in Table 1 and Figure 2 and Figure 3 demonstrate a close relationship between blend composition, solution properties, electrospinning behavior, and final fiber morphology.

#### 3.1.3. FTIR and XRD Analysis of Zein/PVDF Nanofiber Membranes

**Figure 4:**
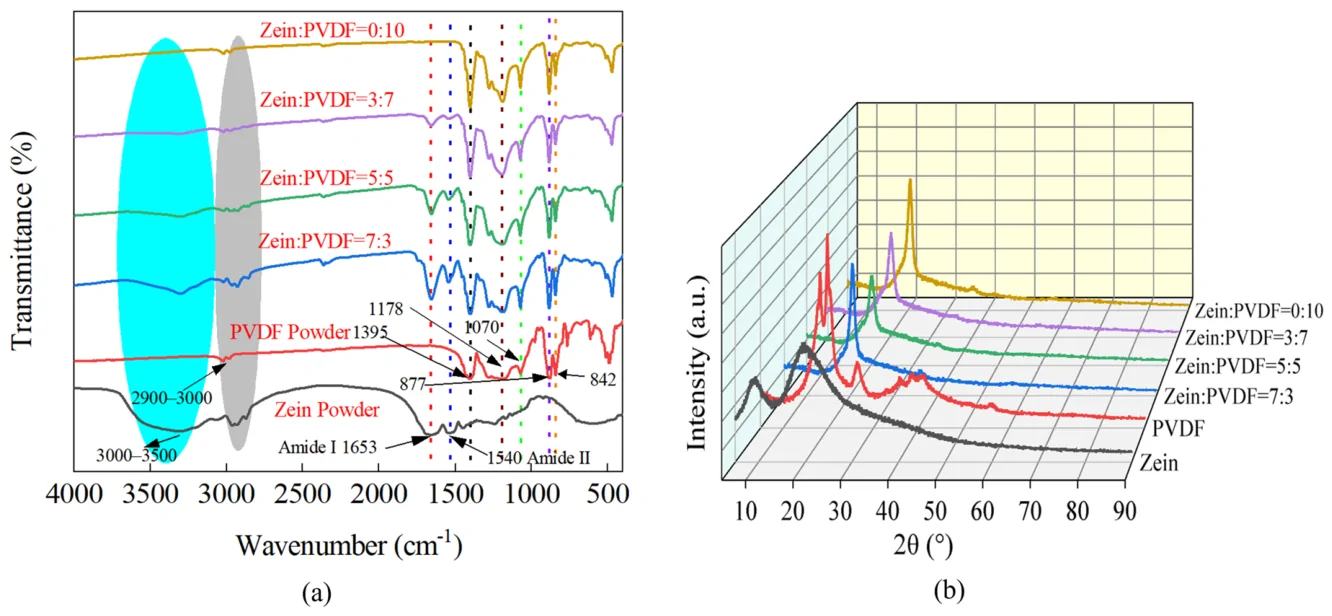
FTIR spectra (**a**) and XRD patterns (**b**) of raw Zein and PVDF powders used as reference materials and Zein/PVDF electrospun samples. Major characteristic FTIR bands are labeled with their corresponding wavenumbers in panel (**a**).

Figure 4a shows the FTIR spectra of pure Zein, pure PVDF, and Zein/PVDF composite nanofiber membranes prepared at different mass ratios. Pure Zein exhibited a broad absorption region at 3000–3500 cm^−1^ associated with N–H and O–H stretching, together with characteristic amide I and amide II bands at approximately 1653 and 1540 cm^−1^, respectively [26,27]. In contrast, pure PVDF displayed prominent bands at approximately 1395, 1178, 1070, 877, and 842 cm^−1^, which are associated with characteristic –CH_2_–/–CF_2_– and C–F vibrations of PVDF [28]. A weak region around 2900–3000 cm^−1^ was also associated with C–H stretching. With increasing PVDF content, the PVDF-related bands became progressively more pronounced, whereas the characteristic amide bands of Zein weakened. The coexistence of Zein- and PVDF-related absorption bands in the composite membranes confirms successful incorporation of both components. No new absorption bands were observed, suggesting that the two polymers were combined primarily through physical blending and intermolecular interactions rather than through the formation of new covalent bonds.

Figure 4b shows the XRD patterns of the Zein, PVDF, and composite nanofiber membranes. Pure Zein exhibited broad and weak diffuse peaks at approximately 2θ = 22.5°, indicating its predominantly amorphous nature [29], whereas pure PVDF exhibited distinct crystalline diffraction peaks, confirming its typical semi-crystalline characteristics [30]. For the Zein/PVDF composite nanofiber membranes, both the broad amorphous peak of Zein and the characteristic crystalline peaks of PVDF were retained, indicating the coexistence of the two components in the composite system. Moreover, the diffraction intensity associated with PVDF increased gradually with PVDF content.

As the PVDF content increased, the PVDF-related diffraction peaks became progressively stronger and sharper, whereas the amorphous scattering background of Zein gradually weakened. This result further confirms the coexistence of the two phases and the tunable composition of the composite membrane. From a structure–property perspective, the enhanced crystalline peaks of PVDF suggest an increase in the proportion of ordered crystalline regions and more regular molecular chain packing, which is consistent with the previously observed increase in the fiber diameter with increasing PVDF content. Meanwhile, the composite membranes retained certain amorphous characteristics, indicating that the introduction of Zein preserved the amorphous phase of the system to some extent and may have contributed to improved flexibility and interfacial compatibility.

#### 3.1.4. Thermal Stability Analysis of Zein/PVDF Nanofiber Membranes

**Figure 5:**
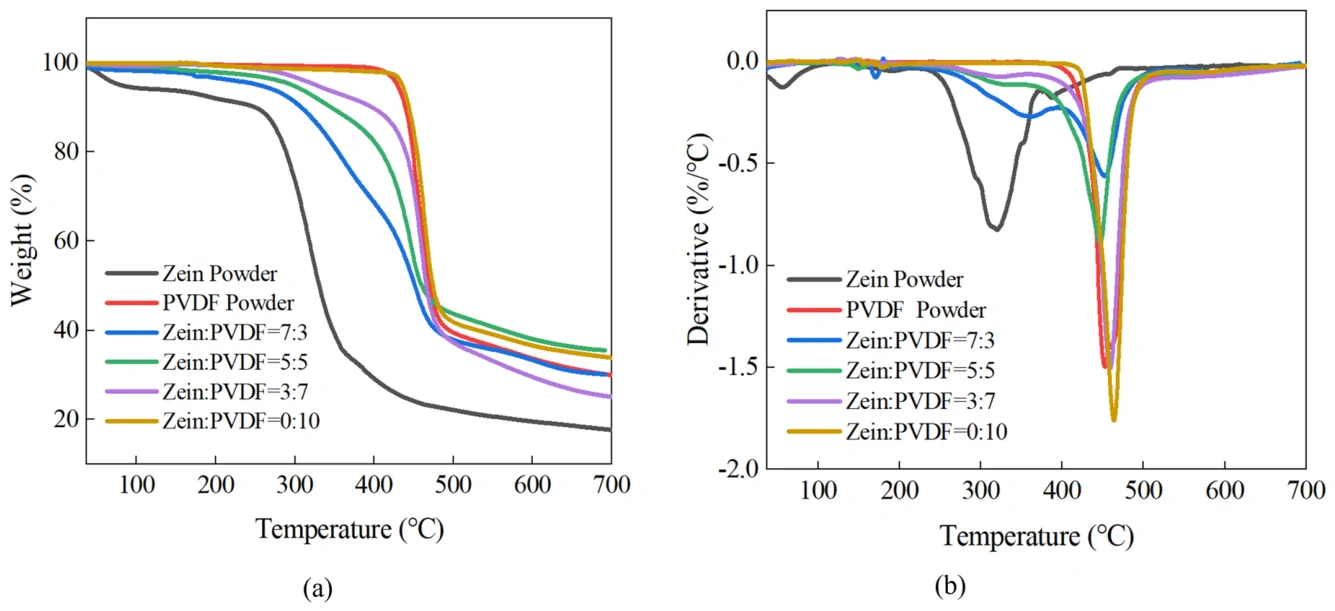
TG (**a**) and DTG (**b**) curves of raw Zein and PVDF powders used as reference materials and Zein/PVDF electrospun samples.

Figure 5 presents the TG and DTG curves of Zein/PVDF nanofiber membranes with different mass ratios. Pure Zein exhibited slight weight loss at approximately 100–150 °C, mainly due to the evaporation of adsorbed water and residual solvent, followed by major thermal degradation over 250–380 °C. The broad DTG peak in this region suggests overlapping degradation processes associated with the decomposition of the protein structure, including peptide bond cleavage and degradation of amino acid residues [31]. In contrast, pure PVDF showed little weight loss below approximately 430 °C and underwent rapid decomposition mainly within 450–520 °C, producing a sharp DTG peak characteristic of its dominant thermal degradation stage. A similar degradation range has been reported for electrospun PVDF fibers [32], supporting the higher thermal stability of PVDF relative to Zein.

The Zein/PVDF composite membranes exhibited thermal degradation characteristics of both components, with the major weight loss occurring mainly within 300–520 °C. The shoulder or relatively broad degradation region at lower temperatures was associated primarily with Zein decomposition, whereas the sharp peak at approximately 470–500 °C was attributed mainly to PVDF degradation. As the PVDF content increased, the dominant degradation region gradually shifted toward the characteristic decomposition temperature of PVDF, indicating an increasing contribution of PVDF to the thermal behavior of the composite membranes. Similar composition-dependent changes in thermal stability have also been reported for PVDF-based electrospun polymer blends [33].

The slight shift in the Zein-associated degradation region toward higher temperatures, together with the reduced DTG peak intensity, suggests that the incorporation of PVDF altered the thermal degradation behavior of Zein, possibly through intermolecular interactions and interfacial constraints within the blended matrix. Overall, increasing the PVDF fraction improved the medium- and high-temperature thermal stability of the Zein/PVDF membranes, consistent with the intrinsically higher thermal resistance of PVDF.

#### 3.1.5. Surface Wettability, Water Vapor Permeability, and Water Solubility of Zein/PVDF Nanofiber Membranes

**Figure 6:**
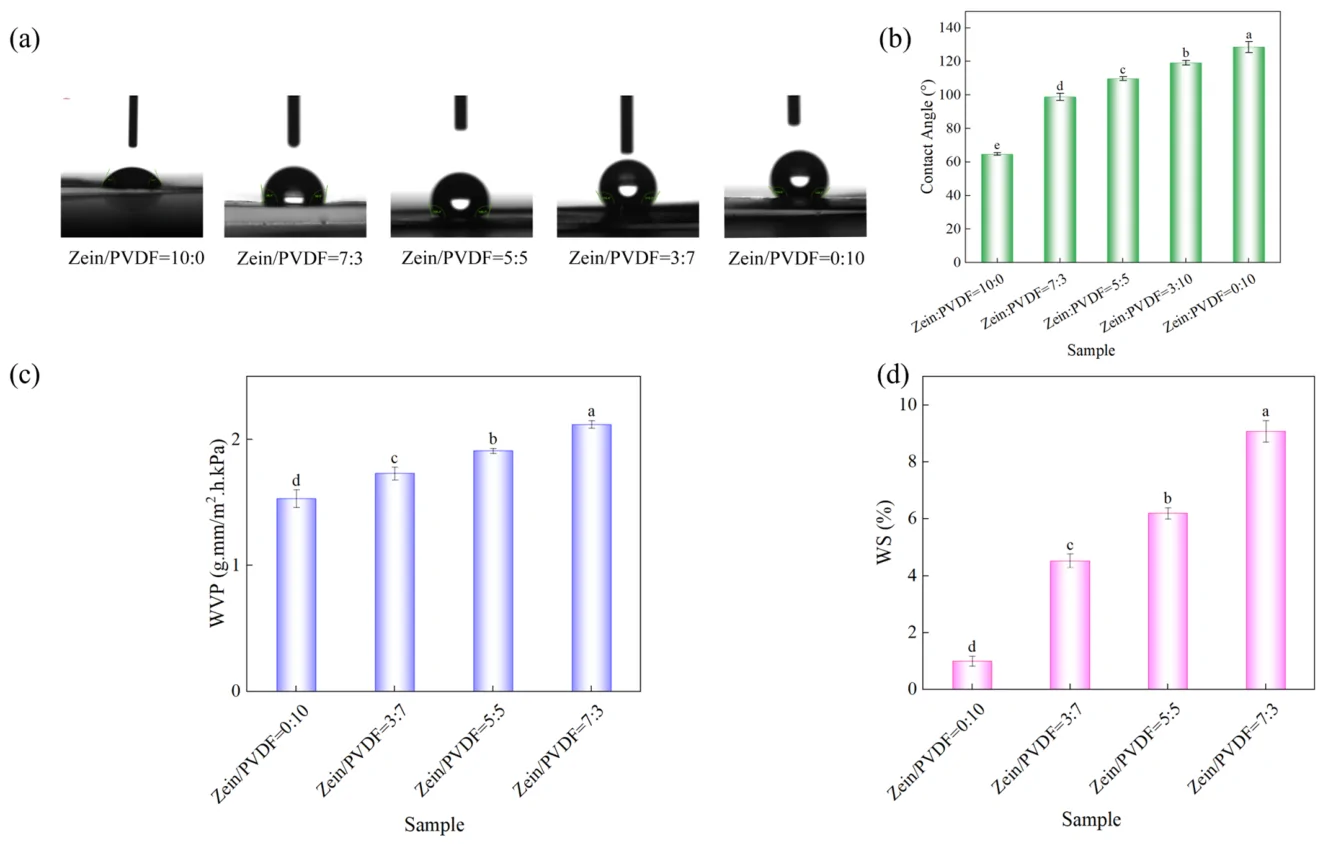
Surface wettability, water vapor permeability, and water solubility of Zein/PVDF composite nanofiber membranes with different mass ratios: (**a**) water contact angle images; (**b**) water contact angle values; (**c**) water vapor permeability (WVP); and (**d**) water solubility (WS). Data in (**b**–**d**) are expressed as mean ± SD. Different lowercase letters indicate significant differences among samples according to one-way ANOVA followed by Duncan’s multiple range test (*p* < 0.05).

Figure 6 shows the effects of different Zein/PVDF mass ratios on the surface wettability, water vapor permeability (WVP), and water solubility (WS) of the composite membranes. As the PVDF content increased, the water contact angle gradually increased from approximately 63.5° for the Zein/PVDF = 10:0 sample to approximately 130° for pure PVDF, indicating a marked enhancement in surface hydrophobicity. The contact angle of pure PVDF was close to the approximately 131° reported previously for electrospun PVDF nanofibrous membranes [32]. This behavior was mainly attributed to the low surface energy of the –CF_2_– groups in PVDF, while the micro/nanoscale roughness generated during electrospinning may further enhance the apparent hydrophobicity of the fibrous surface [34].

In contrast, both WVP and WS increased with increasing Zein content. This behavior can be attributed to the abundant –OH, –NH, and amide groups in Zein, which enhance membrane–water interactions, promote water sorption, and facilitate water vapor transport. The stronger water affinity of Zein-rich membranes may also promote swelling or partial dissolution, thereby increasing WS. Previous studies have shown that the moisture sorption behavior and WVP of Zein films are strongly related to their composition and environmental relative humidity [35]. Moreover, WVP is known to be affected by environmental conditions such as relative humidity [22]. These findings support the observed increase in WVP with increasing Zein content and highlight the important role of polymer–water interactions in water vapor transport.

Overall, increasing the PVDF content enhanced membrane hydrophobicity and water resistance, whereas increasing the Zein content favored water sorption and water vapor transport. Considering spinnability, fiber morphology, mechanical performance, and water-related properties, the Zein/PVDF = 5:5 formulation exhibited a comparatively balanced overall performance and was therefore selected as the carrier matrix for subsequent functionalization.

#### 3.1.6. Mechanical Properties Analysis of Zein/PVDF Nanofiber Membranes

**Figure 7:**
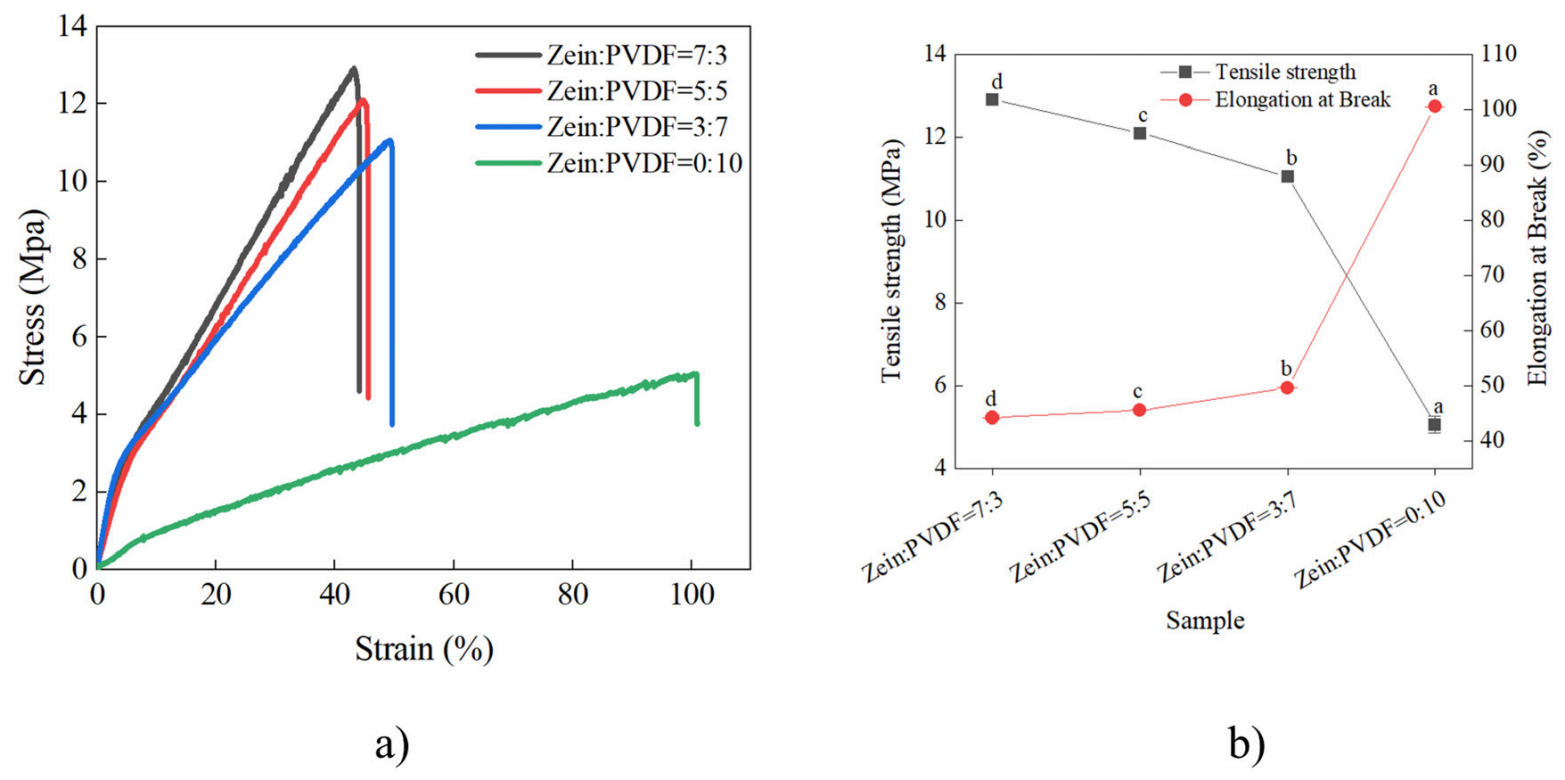
Stress–strain curves (**a**) and tensile strength and elongation at break (**b**) of Zein/PVDF composite nanofiber membranes with different mass ratios. Data in (**b**) are expressed as mean ± SD. Different lowercase letters indicate significant differences among samples according to one-way ANOVA followed by Duncan’s multiple range test (*p* < 0.05).

Figure 7 shows the stress–strain behavior, tensile strength, and elongation at break of the Zein/PVDF composite nanofiber membranes. As shown in Figure 7a,b, increasing the PVDF content produced a clear strength–ductility trade-off. The tensile strength decreased from approximately 13 MPa for the Zein/PVDF = 7:3 membrane to approximately 5 MPa for pure PVDF, whereas the elongation at break increased from approximately 42% to approximately 100%. The 5:5 and 3:7 formulations exhibited intermediate tensile strengths of approximately 12 and 11 MPa, respectively, with elongation values of approximately 43% and 48%. These composition-dependent changes are consistent with the general behavior reported for electrospun protein/polymer systems, in which fiber architecture and polymer composition strongly influence load transfer and extensibility [2,36]. From a structural perspective, the smaller-diameter fibers in Zein-rich membranes can form denser networks with greater inter-fiber contact, restricting fiber slippage and favoring higher tensile strength. In contrast, increasing PVDF content promoted polymer-chain mobility and deformation, thus increasing ductility, while the simultaneous increase in fiber diameter and reduction in effective inter-fiber load transfer contributed to lower tensile strength. Because mechanical properties of electrospun membranes are strongly affected by membrane thickness, fiber morphology, polymer composition, and test conditions, direct numerical comparison with values reported for other systems should be made cautiously. Overall, the results confirm that the Zein/PVDF ratio provides an effective means of tuning the balance between strength and extensibility.

#### 3.1.7. pH-Responsive Behavior and Freshness-Indicating Performance of BTB/Zein/PVDF Membranes

**Figure 8:**
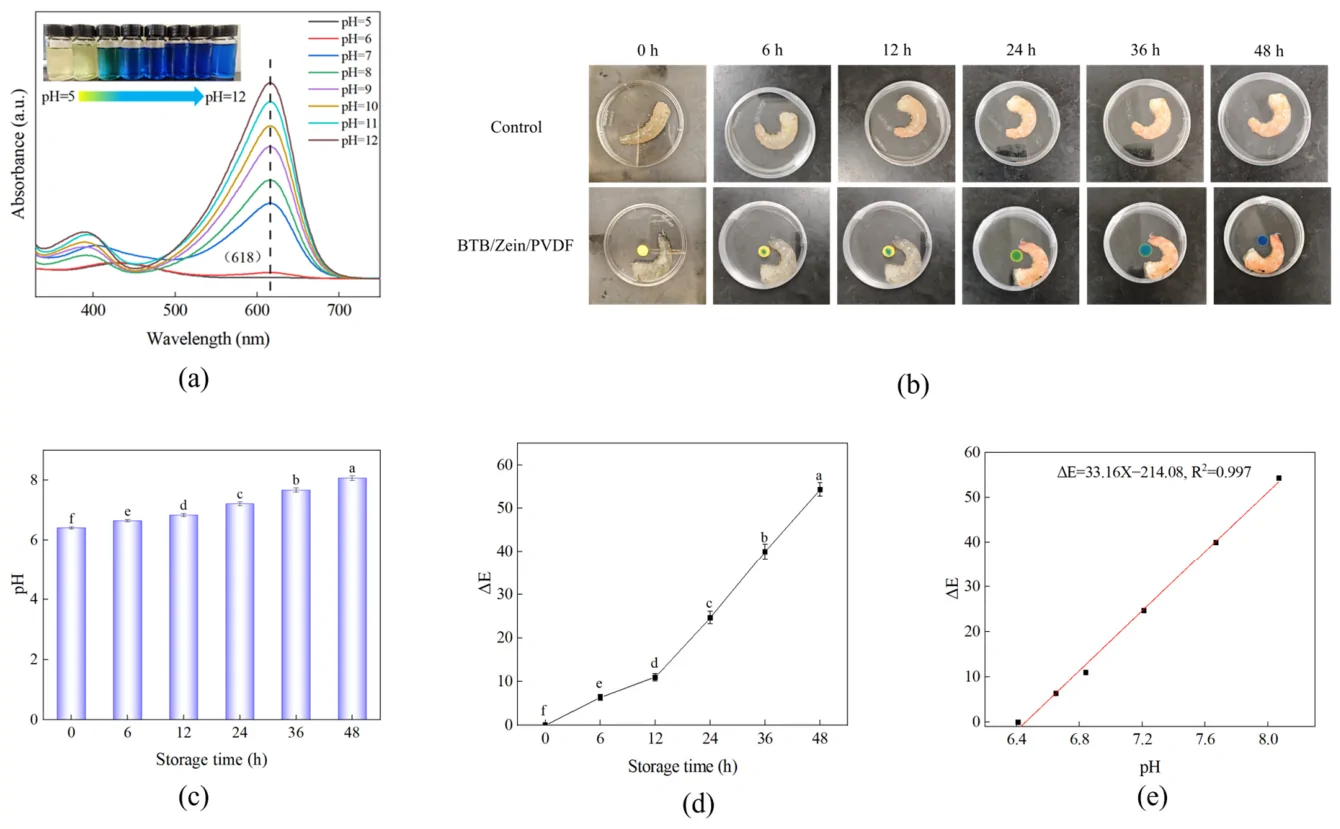
pH-responsive performance of BTB/Zein/PVDF electrospun indicator membranes and their application in shrimp freshness monitoring. (**a**) UV–vis absorption spectra and color changes under different pH conditions; (**b**) representative visual changes in shrimp and the indicator membrane during storage; (**c**) changes in shrimp pH during storage; (**d**) changes in the color difference (ΔE) of the indicator membranes during storage; and (**e**) linear correlation between ΔE and shrimp pH. Data in (**c**,**d**) are expressed as mean ± SD. Different lowercase letters indicate significant differences among storage times according to one-way ANOVA followed by Duncan’s multiple range test (*p* < 0.05).

Figure 8 presents the pH-responsive performance of the BTB/Zein/PVDF indicator membrane and its application in shrimp freshness monitoring. As shown in Figure 8a, the pH-sensitive behavior of BTB was retained after incorporation into the Zein/PVDF fibrous matrix. As the pH increased from acidic to alkaline conditions, the membrane color gradually changed from yellow to green, blue-green, and finally blue, accompanied by an increase in the characteristic absorption band at approximately 618 nm. The corresponding changes in visible color and UV–vis absorption indicate that the Zein/PVDF fibrous matrix effectively retained the acid–base responsiveness of BTB.

The response of the indicator membrane was further evaluated during shrimp storage at 25 °C (Figure 8b–e). The measured pH of the shrimp gradually increased from approximately 6.4 to 8.0 during storage (Figure 8c), reflecting progressive freshness deterioration. Meanwhile, the indicator membrane changed from light yellow/yellow-green to blue-green and became distinctly blue at 48 h (Figure 8b), while the color difference (ΔE) increased progressively with storage time (Figure 8d). The color change in the indicator membrane may be associated with spoilage-related volatile basic compounds released during shrimp deterioration, which altered the local acid–base environment of BTB. Further correlation analysis showed a strong linear relationship between ΔE and the measured shrimp pH (Figure 8e), described by ΔE = 33.16X − 214.08 with R^2^ = 0.997. This close correlation indicates that the color response of the BTB/Zein/PVDF membrane was closely associated with freshness deterioration during shrimp storage.

## 4. Conclusions

This study systematically investigated the effects of the Zein/PVDF ratio on the solution properties, electrospinning behavior, structure, and performance of composite nanofiber membranes. Increasing the PVDF content decreased solution conductivity while increasing viscosity, thereby improving jet stability and promoting the formation of continuous fibrous networks. Variations in blend composition also affected fiber morphology, thermal stability, surface wettability, water-related properties, and mechanical performance. Increasing PVDF content enhanced surface hydrophobicity and water resistance, whereas increasing Zein content favored water vapor transport and water solubility. The mechanical properties exhibited a clear strength–ductility trade-off. Considering these characteristics collectively, the Zein/PVDF = 5:5 formulation exhibited a comparatively balanced overall performance and was selected as the carrier matrix for subsequent functionalization. Incorporation of bromothymol blue (BTB) endowed the optimized membrane with pH-responsive colorimetric behavior, and its color response showed a strong correlation with the measured shrimp pH during storage (R^2^ = 0.997). Overall, these findings establish the composition–solution–fiber–structure–performance relationship of Zein/PVDF electrospun membranes and demonstrate the feasibility of the optimized matrix as a functional carrier for freshness-responsive food-packaging applications.

## Figures and Tables

**Table 1:** Properties of Zein/PVDF spinning solutions with different mass ratios.

Sample	Surface Tension (mN/m)	Conductivity (μS/cm)
Zein:PVDF = 10:0	38.89 ± 0.80 ^a^	430 ± 0.70 ^e^
Zein:PVDF = 7:3	37.59 ± 0.21 ^a^	344 ± 1.20 ^d^
Zein:PVDF = 5:5	37.88 ± 0.49 ^a^	277 ± 1.58 ^c^
Zein:PVDF = 3:7	39.58 ± 0.24 ^a^	206.5 ± 1.06 ^b^
Zein:PVDF = 0:10	39.18 ± 0.57 ^a^	8.95 ± 0.03 ^a^

Note: Data are expressed as mean ± SD. Different lowercase letters within the same column indicate significant differences among samples according to one-way ANOVA followed by Duncan’s multiple range test (*p* < 0.05).

## Data Availability

The original contributions presented in the study are included in the article; further inquiries can be directed to the corresponding authors.

## References

[1] Agarwal S., Greiner A., Wendorff J.H. (2009). Electrospinning of Manmade and Biopolymer Nanofibers—Progress in Techniques, Materials, and Applications. Adv. Funct. Mater..

[2] Yao F., Gao Y., Chen F., Du Y. (2022). Preparation and properties of electrospun peanut protein isolate/poly-l-lactic acid nanofibers. LWT.

[3] Luraghi A., Peri F., Moroni L. (2021). Electrospinning for drug delivery applications: A review. J. Control. Release.

[4] Su W., Chang Z., E Y., Feng Y., Yao X., Wang M., Lei F. (2024). Electrospinning and electrospun polysaccharide-based nanofiber membranes: A review. Int. J. Biol. Macromol..

[5] Wang Y., Khan M.A., Chen K., Zhang L., Chen X. (2022). Electrospinning of Natural Biopolymers for Innovative Food Applications: A Review. Food Bioprocess Technol..

[6] Abdelgawad A.M., Hudson S.M., Rojas O.J. (2014). Antimicrobial wound dressing nanofiber mats from multicomponent (chitosan/silver-NPs/polyvinyl alcohol) systems. Carbohydr. Polym..

[7] Huang C., Thomas N.L. (2019). Fabrication of porous fibers via electrospinning: Strategies and applications. Polym. Rev..

[8] Mendes A.C., Stephansen K., Chronakis I.S. (2017). Electrospinning of food proteins and polysaccharides. Food Hydrocoll..

[9] Zheng L., Zhu Z., Pan L., Zhong L., Xiao S., Zhao C., Zhang Y. (2025). Polysaccharides and proteins as natural polymers for electrospun wound dressings: A review of healing potential, challenges, and crosslinking strategies. Int. J. Biol. Macromol..

[10] da Popov Pereira Cunha M.D., Aldana A.A., Abraham G.A. (2021). Photo-crosslinked soy protein-based electrospun scaffolds. Mater. Lett. X.

[11] Wei L., Yang Y., Qin Z., Liang F., Xie H. (2025). Fabrication of polyvinyl alcohol/soy protein isolate-based composite nanofilm for preserving Chinese cabbage. Food Chem. X.

[12] Deng L., Zhang X., Li Y., Que F., Kang X., Liu Y., Zhang H. (2018). Characterization of gelatin/zein nanofibers by hybrid electrospinning. Food Hydrocoll..

[13] Shi C., Xi S., Han Y., Zhang H., Liu J., Li Y. (2019). Structure, rheology and electrospinning of zein and poly(ethylene oxide) in aqueous ethanol solutions. Chin. Chem. Lett..

[14] Ullah S., Hashmi M., Shi J., Kim I.S. (2023). Fabrication of Electrospun PVA/Zein/Gelatin Based Active Packaging for Quality Maintenance of Different Food Items. Polymers.

[15] Zhang Y., Yang K., Qin Z., Zou Y., Zhong H., Zhang H. (2022). Cross-linked gluten/zein nanofibers via Maillard reaction with the loading of star anise essential oil/β-cyclodextrin inclusions for food-active packaging. Food Packag. Shelf Life.

[16] Valizadeh R., Zandi M., Ganjloo A., Dardmeh N. (2024). Electrospun zein fibers loaded with pomegranate flower extract: Characterization and release behavior. Food Biosci..

[17] Zhan F., Yan X., Sheng F., Li B. (2020). Facile in situ synthesis of silver nanoparticles on tannic acid/zein electrospun membranes and their antibacterial, catalytic and antioxidant activities. Food Chem..

[18] Gopi S., Balakrishnan P., Pius A., Thomas S. (2017). Chitin nanowhisker (ChNW)-functionalized electrospun PVDF membrane for enhanced removal of Indigo carmine. Carbohydr. Polym..

[19] Rajamohan R., Muhammed A.P., Raorane C.J., Ramasundaram S., Raja I.S., Subramanian S.A., Sun S. (2025). Electrospun Nanofibers of Polyvinylidene Fluoride Enriched with Active Antimicrobial Tannic Acid for the Improvement of the Shelf Life of Cherry Tomatoes. Materials.

[20] Teng D., Wahid A., Zeng Y. (2020). Zein/PVDF micro/nanofibers with improved mechanical property for oil adsorption. Polymer.

[21] Gontard N., Guilbert S., Cuq J.-L. (1992). Edible Wheat Gluten Films: Influence of the Main Process Variables on Film Properties using Response Surface Methodology. J. Food Sci..

[22] McHugh T.H., Avena-Bustillos R., Krochta J.M. (1993). Hydrophilic Edible Films: Modified Procedure for Water Vapor Permeability and Explanation of Thickness Effects. J. Food Sci..

[23] Yao F., Gao Y.-H., Chen F.-S., Xia Y.-M. (2021). Effects of electrospinning parameters on peanut protein isolate nanofibers diameter. CyTA-J. Food.

[24] Mukka S., Ziegler G.R. (2024). Aligned electrospun starch-pullulan-protein fibers. Food Hydrocoll..

[25] Zhao Y.-M., Li Y., Ma H., He R. (2023). Effects of ultrasonic-assisted pH shift treatment on physicochemical properties of electrospinning nanofibers made from rapeseed protein isolates. Ultrason. Sonochem..

[26] İnan-Çınkır N., Ağçam E., Altay F., Akyıldız A. (2024). Emulsion electrospinning of zein nanofibers with carotenoid microemulsion: Optimization, characterization and fortification. Food Chem..

[27] Ramos-Souza C., da Trindade L.G., Braga A.R.C., Perrechil F., De Rosso V.V. (2025). Electrospun microfibers of zein and polyethylene oxide: A study on pequi carotenoid encapsulation. Food Hydrocoll..

[28] Lee J.C., Suh I.W., Park C.H., Kim C.S. (2021). Polyvinylidene fluoride/silk fibroin-based bio-piezoelectric nanofibrous scaffolds for biomedical application. J. Tissue Eng. Regen. Med..

[29] Ma Y., Li X., Cai L., Li J. (2022). pH-Sensitive ε-polylysine/polyaspartic acid/zein nanofiber membranes for the targeted release of polyphenols. Food Funct..

[30] Guo J., Chen B., Shen Y., Chen C., Yang Y., Yu D. (2024). Morphological Modulation of Electrospun PVDF/PVP Nanofibers. J. Polym. Sci..

[31] Dhandayuthapani B., Varghese S.H., Aswathy R.G., Yoshida Y., Maekawa T., Sakthikumar D. (2012). Evaluation of Antithrombogenicity and Hydrophilicity on Zein-SWCNT Electrospun Fibrous Nanocomposite Scaffolds. Int. J. Biomater..

[32] Yalcinkaya F., Yalcinkaya B., Pazourek A., Mullerova J., Stuchlik M., Maryska J. (2016). Surface Modification of Electrospun PVDF/PAN Nanofibrous Layers by Low Vacuum Plasma Treatment. Int. J. Polym. Sci..

[33] Mustfa W.M., Habeeb S.A. (2023). Evaluation of the Physical Properties and Filtration Efficiency of PVDF/PAN Nanofiber Membranes by Using Dry Milk Protein. Mater. Res. Express.

[34] Li J., Fu J., Cong Y., Wu Y., Xue L., Han Y. (2006). Macroporous fluoropolymeric films templated by silica colloidal assembly: A possible route to super-hydrophobic surfaces. Appl. Surf. Sci..

[35] Wang Y., Padua G.W. (2004). Water Sorption Properties of Extruded Zein Films. J. Agric. Food Chem..

[36] Zheng Y., Yao F., Chen F. (2022). Curcumin-loaded electrospun peanut protein isolate/poly-l-lactic acid nanofibre membranes: Preparation and characterisation and release behaviour. LWT.

